# Interface Driven Effects in Magnetization-Induced Optical Second Harmonic Generation in Layered Films Composed of Ferromagnetic and Heavy Metals

**DOI:** 10.3390/ma14133573

**Published:** 2021-06-26

**Authors:** Evgeniy Mamonov, Irina Kolmychek, Victoria Radovskaya, Igor Pashen’kin, Nikita Gusev, Anton Maydykovskiy, Marina Temiryazeva, Alexei Temiryazev, Tatiana Murzina

**Affiliations:** 1 Department of Physics, M. V. Lomonosov Moscow State University, Leninskie Gory, 1, 62, 119991 Moscow, Russia; mamonov@shg.ru (E.M.); irisha@shg.ru (I.K.); www-rviktoria@ya.ru (V.R.); anton@shg.ru (A.M.); 2 Institute for Physics of Microstructures RAS, GSP-105, 603950 Nizhny Novgorod, Russia; pashenkin@ipmras.ru (I.P.); gusev@ipmras.ru (N.G.); 3 Kotel’nikov Institute of Radioengineering and Electronics of RAS, Fryazino Branch, Vvedensky Sq. 1, 141190 Fryazino, Russia; mtemiryazeva@gmail.com (M.T.); temiryazev@gmail.com (A.T.)

**Keywords:** second harmonic generation, magnetic thin films, inhomogeneous magnetization

## Abstract

Properties of nanolayers can substantially differ from those of bulky materials, in part due to pronounced interface effects. It is known that combinations of layers of heavy and ferromagnetic metals leads to the appearance of specific spin textures induced by interface-induced Dzyaloshinskyi–Moria interaction (DMI), which attracts much interest and requires further studies. In this paper, we study magneto-optical effects in two- and three-layer films composed of a few nanometer thick Co layer adjacent to nanofilms of non-magnetic materials (Pt, W, Cu, Ta, MgO). For experimental studies of the interface magnetization-induced effects, we used the optical second harmonic generation (SHG) technique known for its high sensitivity to the symmetry breaking. We found that the structural asymmetry leads to the increase of the averaged SHG intensity, as well as to the magnetic field-induced effects in SHG. Moreover, by choosing the proper geometry of the experiment, we excluded the most studied linear in magnetization SHG contributions and, thus, succeeded in studying higher order in magnetization and non-local magnetic effects. We revealed odd in magnetization SHG effects consistent with the phenomenological description involving inhomogeneous (gradient) magnetization distribution at interfaces and found them quite pronounced, so that they should be necessarily taken into account when analyzing the non-linear magneto-optical response of nanostructures.

## 1. Introduction

Structuring of a medium at nanoscale can give rise to new effects in their optical and magnetic response [1,2]. It is well recognized nowadays that combinations of thin layers of heavy and ferromagnetic metals demonstrate quite specific spin textures induced to a much extend by interface-induced Dzyaloshinskyi–Moria interaction (DMI) [3,4]. Possible specific magnetic interface patterns are chiral magnetic states, magnetic ripple domains, perpendicular magnetic anisotropy, that can be induced as well by additional ion beam irradiation [5,6], while the most intense interest is attracted to skyrmions considered as promising magnetic quazi-particles with the possibility to control their propagation by external dc electric field [7,8,9]. This bunch of problems requires further studies of properties of interfaces, as well as developing effective techniques for their diagnostics.

The technique of optical second harmonic generation (SHG) is known for its extremely high sensitivity to the state of surfaces and buried interfaces, including morphology, crystallographic symmetry, magnetization state, etc., [10,11]. At the same time, interface-driven effects play a crucial role in the formation of the properties of nanostructures. Moreover, it was demonstrated that the SHG probe allows to reveal specific magnetic ordering, such as antiferromagnetic or vortex states [12,13,14]. Recently, this technique was applied for the investigation of layered structures made of cobalt and heavy metals (Ta, Pt), which were expected to reveal interface Dzyaloshinskyi–Moria interaction; it was shown that besides common magnetooptical effects at the SHG wavelength (MOKE-SHG), extra one intensity effect forbidden for homogeneously magnetized interfaces was observed [15,16]. It was supposed that the underlying mechanism of this effect involves gradient in magnetization terms that can exist at interfaces between magnetic and non-magnetic media. Moreover, a correlation between the values of this “forbidden” effect and of the Dzyaloshinskyi–Moria coefficient was recently observed [16]. In order to study further this non-linear-optical effect, in this work we present a complex analysis of non-linear magneto-optical effects in nanolayered magnetic films composed of the ferromagnetic metal (Co)and a much wider variety of non-magnetic materials (Pt, Ta, W, Cu, MgO).

## 2. Samples and Methods

The films were fabricated by magnetron sputtering of metal or dielectric targets on quartz or glass substrates using a high vacuum magnetron AJA 2200 multichamber system (North Scituate, MA, USA) operating at the basic pressure of 10${}^{-5}$ Pa. Two sets of films were made: (i) two-layer films composed of a 20 nm thick cobalt film and a non-magnetic (NM) layer, Co(20)NM(3), NM = Pt, Ta, W, Cu, MgO; and (ii) three- or four-layer Co-containing fi lms of the composition Pt(3)Co(3)Pt(10), Co(3)Pt(3)Co(10), Co(3)Pt(3)Co(3)Cu(10), Pt(3)Co(3)W(3), the thicknesses of the corresponding layers in nanometers are in the brackets. In order to diminish the possible formation of the in-plane magnetic anisotropy, azimuthal rotation of the substrates was introduced with the angular rate of 25 rounds per minute. Atomic force and magnetic force microscopy studies (MFM) were performed using SmartSPM (AIST-NT, currently produced by HARIBA Scientific, Longjumeau, France), which allowed to reveal the morphology and magnetic structure of the composed films [17].

**Figure 1:**
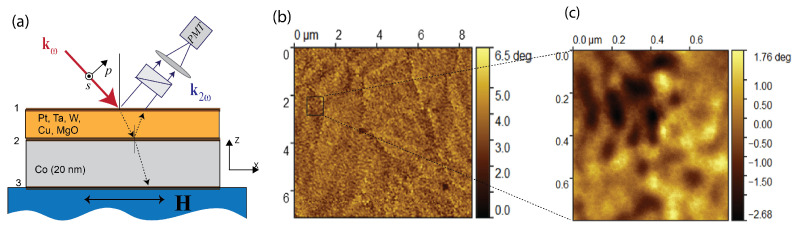
(**a**) Scheme of the experimental configuration for the SHG studies; p- or mixed SHG polarizations were detected, with the magnetic field **H** being parallel to the film surface and to the plane of incidence; (**b**,**c**) magnetic force microscopy images (at different scales) of Co(20)Pt(3) film.

As the fundamental beam in non-linear optical studies we used the radiation of a Ti:Sapphire laser with the wavelength of 820 nm, pulse duration of 20–80 fs, repetition rate of 80 MHz and the mean power of 50 mW focused on the sample into a spot of about 30 μm in diameter. The pump radiation was incident on the sample at the angle of 45${}^{{}^{\circ}}$, SHG radiation reflected from the sample in the direction of specular reflection passed through a necessary set of color filters, Glan-Taylor prism and was detected by a photomultiplier Hamamatsu R4220 (Iwata, Japan) operating in the photon counting mode. In order to diminish the effect of possible laser fluctuations on the measured signal, the reference channel with a crystalline quartz as the SHG source was used with the detection system similar to the signal one; SHG signal from the sample was normalized to that generated by the reference, which increased the signal-to-noise ratio. The scheme of the laser beam interaction with the two-layer film is shown in Figure 1a.

Magneto-optical effects in the SHG response were studied in two geometries: (i) longitudinal non-linear magneto-optical Kerr effect, when the magnetic field-induced rotation of the polarization plane of the SHG wave with respect to initially p-polarization is detected, and (ii) “forbidden” effect, when the longitudinal magnetic field is applied and the magnetization-induced changes of p-polarized SHG was detected. It is worth to remind that the latter effect should vanish for homogeneously magnetized media [10]. For studies of the longitudinal MOKE at the SHG wavelength, the Glan-Taylor analyzer was introduced before the PMT set at 45${}^{{}^{\circ}}$ between s- and p-polarizations, so the polarization plane rotation appeared as the intensity modulation under the application of the dc magnetic field. For both experimental schemes, magnetic field-induced effects in the SHG response can be characterized by the magnetic contrast of the SHG intensity, ρ = $\left(I_{2\omega }\left(+\right)-I_{2\omega }\left(-\right)\right)/\left(I_{2\omega }\left(+\right)+I_{2\omega }\left(-\right)\right)$, where $(I_{2\omega }\left(+\right)$ and $(I_{2\omega }\left(-\right)$ are the SHG intensities measured for the contingently positive and negative longitudinal saturating fields. In the case of common longitudinal magneto-optical SHG effect for the chosen experimental geometry the measured ρ allowed to estimate the angle of the magnetization-induced polarization plane rotation.

## 3. Results

First, the morphology of all the films was studied by atomic force microscopy; it was shown that the mean roughness of the surface is less than 2 nm [16]. At the same time, magnetic force microscopy images (Figure 1b,c) show that in the absence of the external magnetic field the magnetization distribution of Co(20)Pt(3) film is inhomogeneous. On the scale of several microns, domain boundaries are clearly visible (Figure 1b). Inside the domains, one can see a fine magnetic structure with the characteristic scale of about 100 nm (Figure 1c). In static magnetic fields of 20–40 Oe, the movement of domain walls is observed, which disappears if the dc magnetic field is further increased. At the same time, the fine magnetic structure still exists at higher magnetic fields. Thus, on a micron scale, magnetization reversal is typical for films in which shape anisotropy prevails. The fine magnetic structure at the submicron scale may reveal the presence of local anisotropy in polycrystalline films.

**Figure 2:**
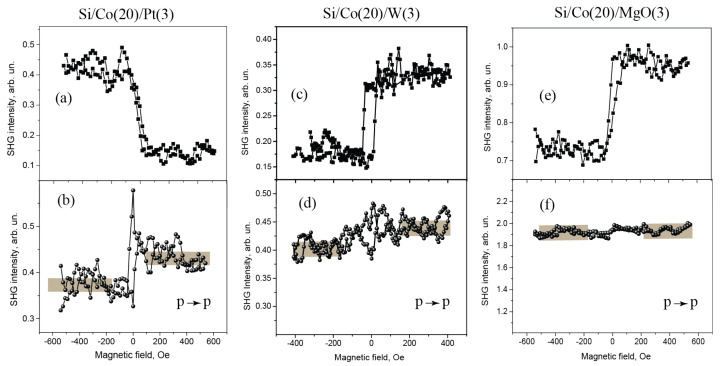
(**a**,**c**,**e**) Magnetic hysteresis loops of the SHG intensity measured in the geometry of allowed MOKE−SHG (mixed SHG polarization) for Si/Co(20)/Pt(3), Si/Co(20)/W(3), and Si/Co(20)/MgO(3) films, respectively; and (**b**,**d**,**f**) magnetic hysteresis loops of the ”forbidden” MOKE−SHG (as the p−polarized SHG intensity was measured).

**Figure 3:**
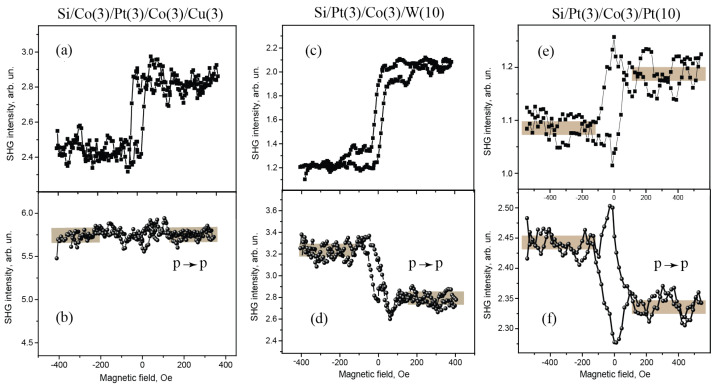
(**a**,**c**,**e**) Magnetic hysteresis loops of the SHG intensity measured in the geometry of allowed MOKE-SHG (mixed SHG polarization) for Si/Co(3)/Pt(3)/Co(3)/Cu(3), Si/Pt(3)/Co(3)/W(10), and Si/Pt(3)/Co(3)/Pt(10), respectively; (**b**,**d**,**f**) similar dependencies for ”forbidden” MOKE−SHG (as the p−polarized SHG intensity was measured) for the same films.

Figure 2 shows the results of comparative studies of magnetic hysteresises of the SHG intensity measured for the longitudinal magneto-optical (“allowed”) Kerr effect (shown in upper panels) and of the “forbidden” effect (the lower ones), for a set of two-layer films with Co layer of 20 nm covered by 3 nm thick layers of different materials. Similar data for some of multilayer 3 nm Co based films are presented in Figure 3.

All the films reveal pronounced magneto-optical longitudinal effect, i.e., induced rotation of the SHG polarization plane that appears as the SHG magnetic contrast ρ; the obtained results for all studied films are summarized in Table 1. As the analyzer was set at 45${}^{{}^{\circ}}$, ρ characterizes the ratio of magnetic to crystallographic components of the SHG polarization, as discussed in the next Section. It is worth noting that the ρ sign for Co-based bilayer films, as well as for all multilayer films, is the same, the only exception being Co(20)Pt(3) one. The highest ρ value (by modulus) is attained for Co(20)Pt(3) and Co(20)W(3), as well as for asymmetric three−layer Pt(3)Co(3)W(3) films. At the same time, for multilayer films that contain two symmetrical magnetic interfaces (e.g., Pt/Co and Co/Pt), the “allowed” magneto-optical SHG effect is low, which reflects the role of interfaces in the observed effects.

Concerning the “forbidden” SHG effect that consists in modulation of the p-polarized SHG intensity by applied longitudinal magnetic field, the observed magnetic contrast in all cases does not exceed a few percent (see Table 1). Among the studied bilayer structures, it is the largest for Co(20)/Pt(3) and Co(20)/W(3) films and is within the experimental error as cobalt is covered by other types of materials like Cu or MgO. This reflects an important role played by the interfaces between a ferromagnetic and heavy metals in the appearance of MOKE−SHG.

Among three-layer Co-containing films, only asymmetric Pt(3)/Co(3)/W(3) shows detectable ρ of the value of 8%, while for the others with more symmetric structures, e.g., Pt(3)/Co(3)/Pt(10) and inverse one, Co(3)/Pt(3)/Co(10), the magnetic contrast of the SHG intensity is within the experimental error. Similarly to the case of longitudinal MOKE-SHG, the films with the largest structural asymmetry reveal the highest magnetic field induced modulation of p-polarized SHG. Importantly that the ρ value remains nearly unchanged as the applied magnetic field is increased far above the saturating values of about 100 Oe (up to 1.5 kOe).

## 4. Discussion

Let us discuss the consequences of the observed effects in the SHG response of studied films of different compositions and in the considered experimental geometries. First it is necessary to remind that due to the inversion symmetry of all the constituent materials used for the fabrication of the films, SHG sources in the electric dipole approximation are attributed to symmetry breaking at the interfaces. This is valid as well in the case of magnetic field induced terms of the non-linear susceptibility that determine magneto-optical effects at the SHG wavelength. Following the description presented in [18,19], the i-th component of the non-linear polarization at the SHG wavelength, which is the source of the SHG wave, can be presented through the so called crystallographic (non-magnetic) and a set of magnetization-sensitive components of non-linear susceptibility as:

(1) $$\begin{array}{c} P_{i}=\left(\chi_{ijk}^{0}+\chi_{ijk}^{M}\right)E_{j}E_{k}, \end{array}$$

where **M** is the magnetization, $\chi^{0}$ and $\chi^{M}$ are the non-magnetic (crystallographic) and magnetization-induced components of the SHG susceptibility, the latter one when taking into account the terms induced by gradient of magnetization can be expressed as:

(2) $$\begin{array}{c} \chi_{ijk}^{M}=\chi_{ijkL}^{(1),M}M_{L}+\chi_{ijkLM}^{(2),M}M_{L}M_{M}+\chi_{ijknM}^{(3),\nabla M}\nabla_{n}M_{M}+\chi_{ijkLnM}^{(4),\nabla M}M_{L}\nabla_{n}M_{M}... \end{array}$$

The first term in (2) describes common magneto-optical Faraday and Kerr effects at the SHG wavelength, which are odd in magnetization. The second one is responsible for even in **M** non-linear optical effects, while the last terms appear if the magnetization distribution is inhomogeneous. It is worth noting that $\chi^{(4),\nabla M}$ is allowed in the bulk of centrosymmetric media and, thus, should dominate.

According to the symmetry analysis first presented in [10], longitudinal MOKE–SHG (when the DC magnetic field is applied along the (OX) axis, the relevant coordinate frame is introduced in Figure 1a) is proportional to the ratio $\chi_{ijkX}^{(1),M}M_{X}/\chi_{ijk}^{0}$. A large value of this effect in the case of the SHG response as compared to linear magneto-optical analogue is typical for the metal magnetic interfaces [11,20]. If so, negative sign of the SHG-MOKE for the Co/Pt bilayer film that differs from the films of other compositions reveals different interface properties (Table 1, first line), while the reason for this requires further studies. It is worth noting that the highest value of the MOKE-SHG is attained for Co/Pt and Co/W interfaces, i.e., as cobalt is adjacent to heavy metals. One can suppose that specific interface ordering intrinsic to such type of interfaces can play a role in the increase of the MOKE-SHG effect.

If considering multilayer films, the strongest magnetic field induced effect in the SHG response is observed only in the case of non-equivalent magnetic interfaces, i.e., Pt/Co and Co/W, coexist in the same structure, while for symmetric ones like Co/Pt/Co or Pt/Co/Pt it is within the experimental error. Strong MOKE-SHG effect for asymmetric three-layer films was reported recently for the transmission geometry [19], while only two structures were analyzed. Here we show that the order of the magnetic interfaces, as well as the integral thickness of the ferromagnetic materials in the film is not important, which also confirms that the main source of the MOKE-SHG is located at magnetic interfaces.

Similar conclusion on the role of interfaces in the appearance of MOKE-SHG effects in Co-containing films stems from the analysis of the value of the “forbidden” effect in various structures. First of all it is necessary to remind that for the case of longitudinal magnetic field only induced rotation of the SHG polarization plane is expected for homogeneously magnetized surfaces, so the intensity of the p-polarized SHG detected in the experiment should remain unchanged [10]. This contradicts with the results of the experiment, as non-zero SHG magnetic contrast ρ is observed in the case of Co/heavy-metal interfaces present in the two-layer structures and in asymmetric Pt/Co/W film. So it was suggested that gradient in magnetization terms like $\chi^{(3),\nabla M}$ and $\chi^{(4),\nabla M}$ can provide the modulation of the SHG intensity [16,19]. At the same time, it stems from the symmetry analysis that odd in longitudinal magnetization terms for in-plane isotropic films can appear only due to $\chi^{(4),\nabla M}$ contributions, as shown in [18,19]. One can suppose that ∇M term in the direction perpendicular to the film, which determines the efficiency of $\chi^{(4),\nabla M}$ contribution to the SHG polarization, increases if the Co layer is squeezed between two non-equivalent heavy metals. This is consistent with the data of the experiments.

## 5. Conclusions

Summing up, we studied experimentally the appearance of magneto-optical effects accompanying second harmonic generation in two- and three-layer films containing ferromagnetic cobalt layers adjacent to different non-magnetic specimen. Besides common longitudinal magneto-optical SHG effect consisting in the magnetic field induced SHG polarization plane rotation, extra one forbidden for homogeneous ferromagnetic interface is observed in a number of layered structures. A set of experiments performed in two- and multilayered films containing ferromagnetic layers shows that this SHG effect is the most pronounced if (i) the structure contains interfaces between ferromagnetic (Co) and heavy metals (e.g., Pt, W), and (ii) is enhanced if the Co-containing multilayer is squeezed between different heavy metals (Pt/Co/W). As a qualitative explanation of the “forbidden” effect in the intensity of the p-polarized SHG induced by the longitudinal magnetic field we suggest inhomogeneous magnetization distribution in the direction perpendicular to the films’ surface.

## Figures and Tables

**Table 1:** Magnetic contrast of the SHG intensity for various planar structures measured in reflection in the scheme of the longitudinal MOKE (p→mix polarization combination) and in the forbidden geometry (p→p polarization combination).

Two-Layer Films					
**Film**	**Co(20)Pt(3)**	**Co(20)Ta(3)**	**Co(20)Cu(3)**	**Co(20)W(3)**	**Co(20)MgO(3)**
p→mix	−28%	+14%	+11%	+28%	+14.5%
p→p	(+6 ± 1.5)%	(+3 ± 1.5)%	(−2 ± 1.5)%	(+4 ± 1.5)%	(+1 ± 2)%
**Three-Layer Films**					
**Film**	**Pt(3)Co(3)Pt(10)**	**Co(3)Pt(3)Co(10)**	**Co(3)Pt(3)Co(3)Cu(10)**	**Pt(3)Co(3)W(3)**	
p→mix	(+4 ± 2)%	(+1 ± 2)%	(+7 ± 1.5)%	(+28 ± 4)%	
p→p	(−2 ± 1.5)%	(−1.5 ± 1.5)%	(+0.5 ± 2)%	(−8 ± 2)%	

## Data Availability

The data presented in this study are available on request from the corresponding author.
